# Mortality in Patients With Hodgkin Lymphoma and Heart Failure: A Swedish Population-Based Study

**DOI:** 10.1016/j.jacadv.2026.103058

**Published:** 2026-07-22

**Authors:** Sissel Johanne Godtfredsen, Sandra Eloranta, Karin E. Smedby, Stefanie Antonilli, Joachim Baech, Daniel Molin, Kristian Kragholm, Tarec C. El-Galaly, Caroline E. Dietrich

**Affiliations:** a Department of Cardiology, Aalborg University Hospital, Aalborg, Denmark; b Department of Medicine Solna, Clinical Epidemiology Division, Karolinska Institutet, Stockholm, Sweden; c Department of Hematology, Karolinska University Hospital, Stockholm, Sweden; d Department of Immunology, Genetics, and Pathology, Cancer Immunotherapy, Uppsala University, Uppsala Akademiska Hospital, Uppsala, Sweden; e Department of Clinical Medicine, Aalborg University, Aalborg, Denmark; f Department of Hematology, Aarhus University Hospital, Aarhus, Denmark; g Department of Molecular Medicine, Aarhus University Hospital, Aarhus, Denmark; h Department of Clinical Epidemiology, Aarhus University Hospital, Aarhus, Denmark; i Department of Hematology, Odense University Hospital, Odense, Denmark

**Keywords:** cardiotoxcitiy, classical Hodgkin lymphoma, heart failure, prognosis

## Abstract

**Background:**

While individuals treated for classical Hodgkin lymphoma (cHL) have an increased risk of heart failure (HF), the effect on survival is less clear.

**Objectives:**

The purpose of this study was to compare all-cause mortality following HF diagnosis between patients with prior cHL and comparators, with cardiovascular (CV)-specific mortality as an explorative outcome.

**Methods:**

Patients with cHL and subsequent HF were matched 1:2 with comparators with HF on sex, birth year, year of HF, and contact type (inpatient/outpatient). Follow-up began at HF and ended upon death or censoring (emigration; December 31, 2023; or maximum 5 years). HRs with 95% CIs were estimated using flexible parametric survival models adjusted for matching variables.

**Results:**

Between 2000 and 2022, 257 patients with cHL and 514 comparators were included. The median age was 73 years, and males predominated (64%). Over a median follow-up of 2.5 years for cHL and 3.3 years for comparators, respectively, 130 (51%) and 192 (37%) died. Lymphoma-related death dominated in the cHL group (40%), while CV death dominated among comparators (42%). All-cause mortality was higher for patients with HF and prior cHL than HF comparators (HR: 1.81; 95% CI: 1.44-2.26). CV-specific mortality did not differ overall (HR: 1.17; 95% CI: 0.78-1.75) except for patients with HF ≥10 years after cHL (HR: 3.13; 95% CI: 1.71-5.73).

**Conclusions:**

cHL was associated with higher all-cause mortality following HF diagnosis. CV-specific mortality was significantly higher for patients with HF ≥10 years after cHL, possibly highlighting the impact of late treatment toxicity, although careful interpretation is warranted given the strong competing risk of lymphoma-related deaths.

The prognosis of heart failure (HF) varies substantially depending on the underlying cause.^1^ For patients diagnosed with classical Hodgkin lymphoma (cHL), anthracycline-based chemotherapy and historical large-field mediastinal radiotherapy have generally been acknowledged as key contributors to an increased risk of developing HF, ranging from 3- to 6-fold compared with the general population and persisting decades after treatment.^2^, ^3^, ^4^, ^5^, ^6^, ^7^

With modern first-line treatments achieving long-term survival exceeding 95% among young patients, cHL is one of the most curable malignancies.^8^^,^^9^ As survival has improved, the population of long-term survivors continues to grow, ultimately bringing treatment-related toxicities to the forefront of survivorship care. Among these, cardiovascular (CV) toxicities have been considered a leading cause of morbidity and mortality.^2^^,^^3^^,^^6^ Although radiotherapy techniques have evolved and chemotherapy protocols increasingly emphasize dose optimization and toxicity reduction, many survivors remain at lifelong risk. Supporting this, increased CV mortality has been demonstrated for cHL populations with cause-specific mortality rates ranging from 2- to over 10-fold higher than the general population, particularly in those treated at younger ages.^10^, ^11^, ^12^ Additionally, it has been suggested that HF among patients with cHL may confer a clinical trajectory distinct from that of HF in the general population.^13^ In a study based on 59 patients with doxorubicin-induced cardiomyopathy in the period of 1982 to 1997, HF after cancer treatment was associated with a poorer prognosis than idiopathic HF or HF with ischemic heart disease etiology.^14^

While previous studies have been instrumental in quantifying the overall burden of CV toxicity among survivors of cHL and in informing management at the time of lymphoma diagnosis and treatment planning, they provide limited information on prognosis among the subset of patients with cHL who go on to develop CV disease. In contrast, studying survival conditional on disease can offer information specifically relevant to the group of patients who develop CV disease after treatment for cHL.^15^ Addressing this gap is important for counseling at the time of HF. Thus, the aim of this study was to assess all-cause mortality following HF in patients with a prior cHL diagnosis compared to matched lymphoma-free comparators.

## Material and methods

### Data sources and study population

This was a register-based matched cohort study utilizing LymphomaBase, a database containing all individuals with a lymphoma diagnosis registered in the Swedish Lymphoma Register 2000 to 2023. The National Swedish Lymphoma Register was established as a nationwide quality-of-care register and contains clinical, diagnostic, and detailed treatment information.^16^ The database also contains 10 age- and sex-matched lymphoma-free comparators from the general population per patient with lymphoma and is linked to information from several national health registers. Comparators were drawn from the Swedish Total Population Register, which covers all individuals residing in Sweden for at least 1 year.^17^ For this study, all patients aged ≥18 years at cHL diagnosis were potentially eligible (n = 4,049), along with the entire comparator cohort (n = 633,993).

From LymphomaBase, we identified 257 patients with cHL and 64,749 comparators with incident HF after cHL diagnosis or original matching date, meaning anyone with an HF diagnosis prior to this was not considered. HF was defined through hospital (inpatient) admissions or outpatient records from the National Patient Register using the World Health Organization’s International Classification of Diseases Revisions version 9 and 10 (Supplemental Table 1). At their HF date, patients with cHL were rematched 1:2 to comparators on sex, birth year, year of HF, and contact type for HF (inpatient or outpatient). Matching was performed using random sampling with replacement.^18^ If the matching was unsuccessful, the tolerance for birth and HF year was relaxed stepwise in 2-year increments to a maximum of ±8 years.

### Outcomes

The main outcome of interest was all-cause death. CV-specific mortality was examined as an exploratory outcome to characterize cause-of-death patterns and generate hypotheses regarding mechanisms underlying previously reported CV late effects among cHL populations. Information on the date and cause of death was retrieved from the National Cause of Death Register.^19^ CV deaths were defined as those in which the main cause of death was attributed to a CV condition (Supplemental Table 1). Non-CV causes of death were reported descriptively and categorized as lymphoma, other nonlymphoma cancer, chronic obstructive pulmonary disease, other lung disease, infection, diabetes mellitus, Alzheimer disease and other dementia, or other cause.

### Baseline characteristics

Information on comorbid conditions acquired any time before the index HF date was retrieved from the National Patient Register and the National Prescribed Drug Register (Supplemental Table 1).^20^ Socioeconomic status was approximated by the highest achieved education level gathered from the Swedish Longitudinal Integrated Database for Health Insurance and Labor Market Studies.^21^ Lymphoma-specific variables, including disease characteristics and treatment given, were gathered from the Swedish Lymphoma Register (Supplemental Table 1).

### Statistical analysis

Follow-up started upon date of HF and ended on date of death, emigration, or administrative censoring (December 31, 2023, or after a maximum of 5 years), whichever came first. Comparators with an incident lymphoma after HF were censored at the date of lymphoma diagnosis (n = 4).

Overall survival was estimated using the Kaplan-Meier method. HRs with 95% CIs were obtained from multivariable flexible parametric proportional hazards models, with 3 degrees of freedom (df) for the baseline rate, comparing all-cause mortality rates between patients with prior cHL and comparators. The multivariable models were adjusted for the matching variables: sex; age at HF; calendar year of HF; and contact type. Age and year were modeled allowing for nonlinear effects using restricted cubic splines with 4 df. For details on model selection and model specification for estimation of standardized survival, please see the Supplemental Appendix.

Estimation of cause-specific HRs followed the same modeling strategy as for all-cause mortality. Additionally, cause-specific cumulative risk of CV death was estimated nonparametrically (using the Aalen–Johansen method) and predicted from flexible parametric nonproportional hazards models including the same variables and interactions as used for standardized overall survival (see Supplemental Appendix). Covariate-pattern-specific predictions were obtained for male and female patients with cHL/comparators aged 60 and 80 years at HF diagnosis, respectively.

As an exploratory outcome, HRs were also estimated to assess the effect of time from cHL to HF by categorizing the cHL population as <2, 2 to <10, and ≥10 years between cHL and HF. Both all-cause and cause-specific mortality was compared to that in the comparators. As a sensitivity analysis, time between cHL and HF was further evaluating in patients with prior cHL alone to enable adjustment for lymphoma-related factors. Details on this analysis can be found in the supplementary material.

All analyses were performed using R version 4.5.0 (R Foundation for Statistical Computing) and Stata version 18 (StataCorp. Stata Statistical Software: Release 18, StataCorp LLC). The study was approved by the Swedish Ethical Review Authority (no. 2019-00242).

## Results

### Baseline characteristics overall

A total of 257 patients with HF and a prior cHL were matched to 514 lymphoma-free comparators with HF (Table 1, Central Illustration). Most HF diagnoses were coded as unspecified (94% in cHL, 88% in comparators). Median age at index HF was 73 years in both groups, and males were overrepresented (64%). CV comorbidity at HF was common overall: slightly less than half had hypertension (44% of cHL group and 48% of comparators), one-fourth had atrial fibrillation, and 1 in 5 had diabetes. Both chronic and acute coronary syndrome and chronic kidney disease were more prevalent among comparators. Median follow-up was 2.5 years for cHL and 3.3 years for comparators.

**Table 1:** Baseline Characteristics of Patients With Heart Failure in Individuals With a Previous Classical Hodgkin Lymphoma Diagnosis and Matched Lymphoma-Free Comparators

	HF Patients With Previous cHL Diagnosis (n = 257)	Comparators With HF (n = 514)
Died during follow-up^a^, n (col %)	130 (50.6)	192 (37.4)
From cardiovascular causes^b^, n (% of deaths)	34 (26.2)	81 (42.2)
From lymphoma, n (% of deaths)	55 (42.3)	0 (0.0)
From nonlymphoma cancers n (% of deaths)	9 (6.9)	39 (20.3)
Chronic obstructive pulmonary disease, n (% of deaths)	5 (3.8)	13 (6.8)
Other lung diseases, n (% of deaths)	3 (2.3)	0 (0.0)
Infections, n (% of deaths)	7 (5.4)	18 (9.4)
Diabetes mellitus, n (% of deaths)	4 (3.1)	6 (3.1)
Alzheimer disease and other dementias, n (% of deaths)	3 (2.3)	6 (3.1)
Other^c^, n (% of deaths)	10 (7.7)	29 (15.1)
Follow-up time (y)^a^, median [IQR]	2.5 [0.8, 5.0]	3.3 [1.5, 5.0]
Age at index HF, median [IQR]	73 [63, 80]	73 [63, 80]
Male sex, n (col %)	164 (63.8)	328 (63.8)
Year of index HF, median [range]	2016 [2011, 2022]	2017 [2011, 2022]
Index HF from inpatient register, n (col %)	165 (64.2%)	330 (64.2%)
HF etiology^d^		
Hypertensive cardiomyopathy	3 (1.2)	12 (2.3)
Dilated cardiomyopathy	8 (3.1)	8 (1.6)
Other cardiomyopathies	4 (1.2)	44 (8.6)
Unspecified	242 (94.2)	450 (87.5)
Education level, n (col %)		
Low (<10 y)	99 (38.5)	186 (36.2)
Intermediate (10-12 y)	102 (39.7)	275 (44.4)
High (>12 y)	52 (20.2)	104 (16.9)
Missing	4 (1.6)	9 (1.5)
Hypertension, n (col %)	104 (40.5)	235 (45.7)
Chronic kidney disease, n (col %)	12 (4.7)	36 (7.0)
Peripheral artery disease, n (col %)	17 (6.6)	36 (7.0)
Ischemic stroke, n (col %)	29 (11.3)	63 (12.3)
Diabetes mellitus, n (col %)	60 (23.3)	111 (21.6)
Atrial fibrillation, n (col %)	70 (27.2)	127 (24.7)
Acute coronary syndrome, n (col %)	46 (17.9)	102 (19.8)
Chronic coronary syndrome, n (col %)	33 (12.8)	82 (16.0)

cHL = classical Hodgkin lymphoma; col = column; HF = heart failure; ICD = International Classification of Diseases; n = number of patients.

a Restricted to the first 5 years following heart failure index date.

b Main cause of death.

c Includes deaths from: DE854, DG12, DG20, DG35, DG41, DG71, DJ690, DK590, DK703, DK746, DL97, DL984, DM878, DN17, DN184, DN185, DN189, DN19, N200; Q20–Q28; R990, R998, R999, V01–Y98, U00–U85.

d Aggregated due to low n in specific subcategories. Includes following diagnoses: Hypertensive cardiomyopathy (ICD-10: I110, I130, I132), dilated cardiomyopathy (ICD-10: I420), other cardiomyopathies (ICD-10: I421, I422, I427, I428, I429), unspecified (ICD-10: I50∗).

### Hodgkin lymphoma characteristics and treatment

Among patients with cHL, the median time from lymphoma to index HF was 3.9 years (Table 2, Supplemental Figure 1). Median age at lymphoma diagnosis was 68 years for patients with cHL and subsequent HF, while it was 41 years for patients without subsequent HF. The median year of cHL diagnosis for the HF subgroup was 2008 and 2013 for the non-HF group. At cHL diagnosis, most of the HF subgroup had advanced stage (65%), nonbulky (88%) disease with Eastern Cooperative Oncology Group performance status I-II (86%). Among those who received chemotherapy, doxorubicin-bleomycin-vinblastine-dacarbazine was the most common regimen (43%). For the HF subgroup, the median cumulative anthracycline dose was 210 mg/m^2^, with 38% receiving more than 200 mg/m^2^. Approximately one-fifth had been treated with radiotherapy.

**Table 2:** Clinical and Treatment Characteristics of Classical Hodgkin Lymphoma Patients With and Without Subsequent Heart Failure

	cHL and New-Onset HF (n = 257)	cHL (n = 3,792)
Age at cHL diagnosis		
Median [IQR]	68 [56, 75]	41 [28, 64]
≤60 years, n (col %)	87 (33.9)	2,695 (71.1)
>60 years, n (col %)	170 (66.1)	1,097 (28.9)
Year of cHL diagnosis		
Median [range]	2008 [2004, 2014]	2,013 [2007, 2018]
Years from cHL to index HF		
Median [IQR]	3.9 [1.2-9.5]	-
ECOG performance status, n (col %)		
0-1	222 (86.4)	3,359 (88.6)
2-4	28 (10.9)	359 (9.5)
Missing	7 (2.7)	74 (2.0)
Ann-Arbor stage, n (col %)		
Limited (I-IIA)	75 (29.2)	1,399 (36.9)
Advanced (IIB-IV)	166 (64.6)	2,204 (58.1)
Missing	16 (6.2)	189 (5.0)
Normal SLD^a^, n (col %)		
Yes	129 (50.2)	1,726 (45.5)
No	87 (33.9)	1,018 (26.8)
Missing	41 (16.0)	1,048 (27.6)
Bulky disease, n (col %)		
Yes	21 (8.2)	594 (15.7)
No	226 (87.9)	2,975 (78.5)
Missing	10 (3.9)	223 (5.9)
Chemotherapy, n (col %)		
ABVD	111 (43.2)	2,186 (57.6)
BEACOPP	15 (5.8)	665 (17.5)
CHOP-like	60 (23.3)	250 (6.6)
Other^b^	17 (6.6)	106 (2.8)
Missing	54 (21.0)	558 (15.5)
Cumulative anthracycline dose		
Median [IQR]	210 [100, 300] mg/m^2^	200 [140, 300] mg/m^2^
0 mg/m^2^	16 (6.2)	98 (2.6)
≤200 mg/m^2^	81 (31.6)	1,751 (46.2)
>200 mg/m^2^	98 (38.1)	1,337 (35.3)
Missing	62 (24.1)	606 (16.0)
Radiotherapy, n (col %)		
Yes	44 (17.1)	1,137 (30.0)
No	146 (56.8)	2,171 (57.3)
Missing	67 (26.1)	484 (12.8)

All clinical characteristics are recorded at cHL diagnosis.

ABVD = doxorubicin-bleomycin-vinblastine-dacarbacine; BEACOPP = bleomycin-etoposide-doxorubicin-cyclophosphamide-vincristine-procarbazine-prednisolone; CHOP = doxorubicin-cyclophosphamide-vincristine-prednisone; ECOG = Eastern Cooperative Oncology Group; SLD = serum level of lactate dehydrogenase; other abbreviations as in Table 1.

a Elevated defined as SLD >3.5 (age at diagnosis [or matching date] 18 to 70), SLD >4.3 (age >70)

b The “other” chemotherapy regimens included treatment with bendamustine, CEOP-21 (cyclophosphamide-etoposide-vincristine), LVP (L-asparaginase-vincristine-prednisolone), chlorambucil, cyclophosphamide, DHAP (dexamethasone-cytarabine-cisplatin), Vadriac, or MOPP (mechlorethamine-vincristine-procarbazine-prednisone).

### All-cause death

**Figure 1:**
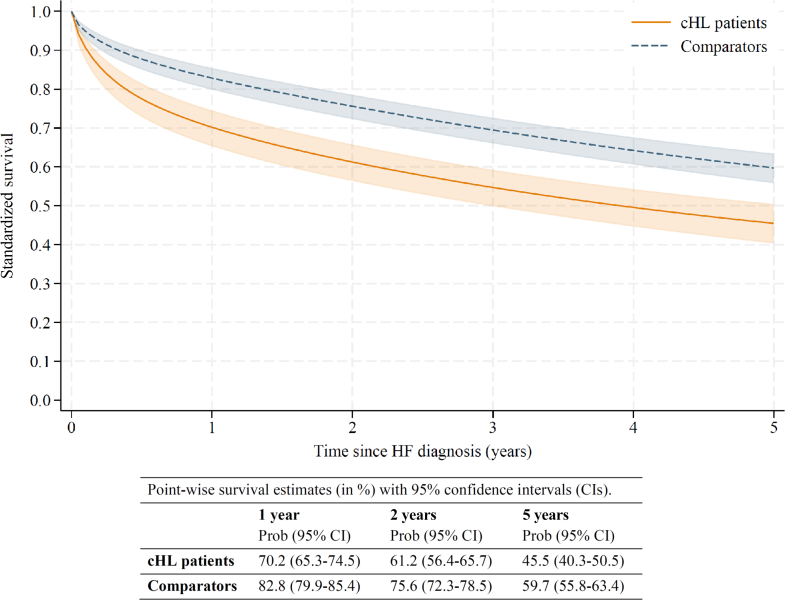
Standardized Overall Survival∗ With 95% CIs Survival after incident heart failure (HF) among patients previously diagnosed with classical Hodgkin lymphoma (cHL) and matched lymphoma-free comparators. Point-wise estimates with uncertainty are given in the accompanying table below. ∗ Predicted from a flexible parametric nonproportional hazards model (with 3 degrees of freedom (df) for the baseline rate and 2 for the time-dependent effect), adjusted for all matching variables: Age and year of HF diagnosis, sex, and source of HF (inpatient/outpatient register). Age and year were modeled assuming nonlinear effects, using restricted cubic splines with 4 df. The effect of exposure to cHL was allowed to vary between sex and age by including interaction terms. Standardized overall adjustment variables. cHL = classical Hodgkin lymphoma; HF = heart failure.

**Figure 2:**
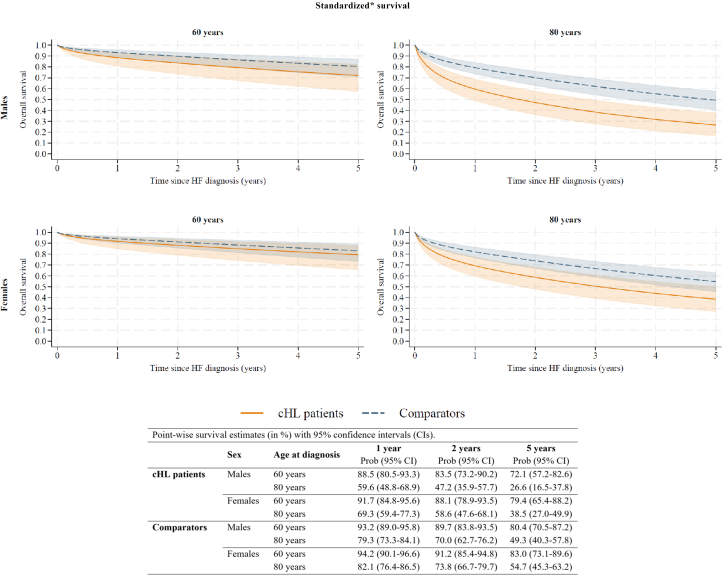
Covariate-Specific Standardized Overall Survival∗ Survival among males (top panel) and females (bottom panel) aged 60 (left) and 80 (right) at heart failure (HF) diagnosis in patients with a previous classical Hodgkin lymphoma (cHL) and matched comparators. Point-wise estimates with uncertainty are given in the accompanying table below. ∗ Predicted from a flexible parametric nonproportional hazards model (with 3 degrees of freedom (df) for the baseline rate and 2 for the time-dependent effect), adjusted for all matching variables: Age and year of HF diagnosis, sex, and source of HF (inpatient/outpatient register). Age and year were modeled assuming nonlinear effects, using restricted cubic splines with 4 df. The effect of exposure to cHL was allowed to vary between sex and age by including interaction terms. Standardized over calendar year and source of HF. Abbreviations as in Figure 1.

During follow-up, 322 deaths were recorded, 130 among cHL (51% of patients) and 192 among comparators (37% of comparators) (Table 1, Supplemental Figure 2). One year after HF, the standardized overall survival was 70.2% for the cHL group (95% CI: 65.3-74.5) and 82.8% for comparators (95% CI: 79.9-85.4) (Figure 1). At 5 years, it was 45.5% (95% CI: 40.3-50.5) in the cHL group and 59.7% (95% CI: 55.8-63.4) among comparators. This corresponded to a nearly 2-fold increased all-cause mortality rate for patients with cHL vs comparators after adjustment for the matching variables (HR: 1.81; 95% CI: 1.44-2.26) (Table 3). In a sensitivity analysis additionally adjusting for history of CV disease and chronic kidney disease, the results remained robust (HR: 1.84; 95% CI: 1.46-2.30, data not shown). Although no evidence of proportional hazards violation was observed (*P* = 0.34 for all-cause mortality), analyses allowing for a time-varying effect indicated that the higher mortality among patients with cHL was most pronounced early after HF and attenuated over time (Supplemental Figure 3). In the model-based predictions examining survival by sex and age at HF diagnosis, the between-group difference was most pronounced in older male patients while differences were more modest in younger and female patients (Figure 2). For a 60-year-old patient, the 5-year standardized survival probability was cHL male: 72.1% (95% CI: 57.2-82.6), comparator male: 80.4% (95% CI: 70.5-87.2), cHL female: 79.4% (95% CI: 65.4-88.2), comparator female: 83.0% (95% CI: 73.1-89.6). For an 80-year-old patient, the corresponding numbers were cHL male: 26.6% (95% CI: 16.5-37.8), comparator male: 49.3% (95% CI: 40.3-57.8), cHL female: 38.5% (95% CI: 27.0-49.9), comparator female: 54.7% (95% CI: 45.3-63.2).

**Table 3:** HRs^a^ With 95% CIs as Measures of Relative All-Cause and Cardiovascular-Specific Mortality Rates Among Patients With HF and a Prior cHL Diagnosis Compared With Matched Lymphoma-Free Comparators With HF

All-Cause Mortality	Events/Person-Years	HR^a^ (95% CI)
cHL patients	130/674.0	1.81 (1.44-2.26)
Comparators	192/1,604.1	1.00 (ref.)
cHL patients		
<2 years since cHL	39/222.7	1.54 (1.08-2.18)
≥2 to <10 years since cHL	63/313.2	1.81 (1.36-2.42)
≥10 years since cHL	28/138.1	2.37 (1.56-3.58)
Comparators	192/1,604.1	1.00 (ref.)
Cardiovascular-specific mortality		
cHL patients	34/674.0^b^	1.17 (0.78-1.75)
Comparators	81/1,604.1^b^	1.00 (ref.)
cHL patients		
<2 years since cHL	5/222.7	0.49 (0.20-1.22)
≥2 to <10 years since cHL	15/313.2	1.05 (0.60-1.83)
≥10 years since cHL	14/138.1	3.13 (1.71-5.73)
Comparators	81/1,604.1	1.00 (ref.)

Estimates are also stratified by time interval between cHL and index HF within the cHL group, contrasted to comparators.

CV = cardiovascular; other abbreviations as in Table 1.

a Estimated from flexible parametric proportional hazards models with 3 degrees of freedom (df) for the baseline hazard rate adjusted for all matching variables: Age and year of HF diagnosis, sex, and source of HF (inpatient/outpatient register). Age and year were modeled assuming nonlinear effects, using restricted cubic splines with four df.

b Note: The person-years reported for CV mortality represent total follow-up time rather than person-time at risk for CV death specifically.

**Central Illustration:**
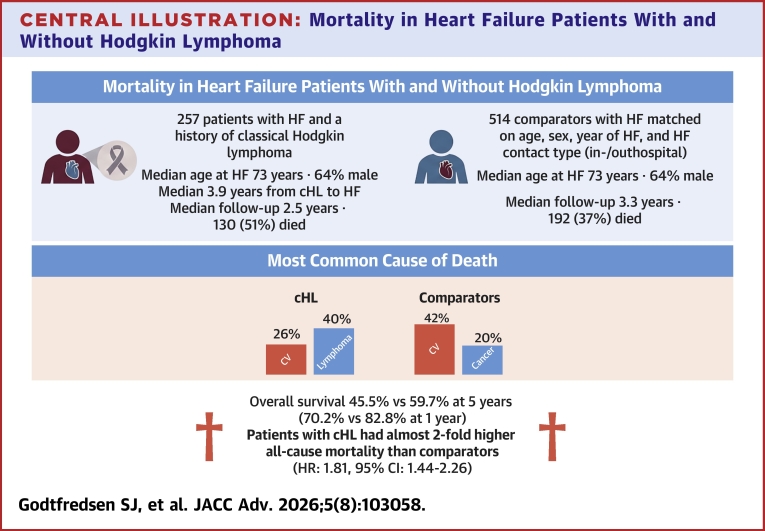
Mortality in Heart Failure Patients With and Without Hodgkin Lymphoma CV = cardiovascular; other abbreviations as in Figure 1.

### Cause-specific death

In the exploratory analyses of cause-specific mortality, the most prevalent cause of death for the cHL group was lymphoma-related (n = 55, 42% of deaths) and secondly CV-related deaths (n = 34, 26%). For the comparators, death due to CV disease was the most common cause (n = 81, 42%) followed by nonlymphoma cancers (n = 39, 20%, Table 1). This did not confer a significant difference in CV-specific mortality between the cHL group and comparators (HR: 1.17; 95% CI: 0.78-1.75, Table 3). There was no evidence of violation of the proportional hazards assumption for CV-specific mortality, neither from formal test (*P* = 0.96) or graphically based on a model allowing for a time-varying effect (Supplemental Figure 3).

In absolute terms, nonparametric estimates of the cumulative risk of CV death were 6.6% (95% CI: 4.0-10.1) and 7.8% (95% CI: 5.7-10.3) at 1 year in patients with cHL and comparators, respectively, increasing to 14.5% (95% CI: 10.3-19.4) and 17.8% (95% CI: 14.4-21.5) at 5 years after HF (Supplemental Figure 4). Model-based predictions of the covariate-specific cumulative risk of CV death did not indicate any significant differences between patients with prior cHL and comparators (Supplemental Figure 5).

### Analyses by time from cHL to index HF

When examining the time interval between cHL diagnosis and index HF, median age at index HF was 74 years for the <2 years group, 74 for 2 to <10 years, and 71 for the ≥10 years group. The cHL groups had progressively higher all-cause mortality than comparators the longer the interval between cHL and HF (adjusted HRs of 1.54 [95% CI: 1.08-2.18] for <2 years between cHL and index HF, 1.81 [95% CI: 1.36-2.42] for 2 to <10 years, and 2.37 [95% CI: 1.56-3.58] for ≥10 years) (Table 3). An analysis modeling the cHL-to-HF interval as a continuous variable using restricted cubic splines (with 4 df) showed a bimodal pattern in the HR for all-cause mortality, with a transient early increase followed by a decrease and a subsequent marked rise beyond approximately 10 years, consistent with the subgroup cutoffs (Supplemental Figure 6).

The distribution of causes of death also changed with increasing interval between cHL and HF; in the <2 and 2- to <10-year groups, the most common cause of death was lymphoma related (24 [62%] and 26 [41%] of deaths for <2 and 2 to <10, respectively), while it was CV disease in the ≥10-year group (14 [50%] of deaths). The corresponding HRs of CV death did not indicate any significant difference to comparators during the first decade (HR < 2 years = 0.49; 95% CI: 0.20-1.22; HR ≥ 2 to <10 years = 1.05; 95% CI: 0.60-1.83) but rose given ≥10 years between cHL and HF (HR: 3.13; 95% CI: 1.71-5.73). When modeling the HR as a function of time from cHL to HF, the CV-specific mortality rate increased just before 10 years and continued to rise throughout (Supplemental Figure 6).

In analyses limited to patients with prior cHL and further adjusting for lymphoma-related factors, increasing time between cHL and HF resulted in higher all-cause mortality: HR ≥2 to 10 years = 1.79 (95% CI: 1.08-2.97) and HR ≥10 years = 3.04 (95% CI: 1.53-6.08) comparing with the <2 years group (Supplemental Table 2). For CV-specific death, the corresponding HRs were 4.50 (95% CI: 1.24-16.3) and 34.6 (95% CI: 7.50-160).

## Discussion

In this population-based study of survival following HF, patients with a prior diagnosis of cHL exhibited worse overall survival compared to individuals without a history of lymphoma. In exploratory analyses, the CV-specific survival was similar between the groups overall, but a longer interval between cHL and HF was associated with increased CV mortality. Taken together, these findings provide valuable insight into the mechanism underlying previously reported CV late effects and related mortality following therapy for cHL, suggesting that treatment effects are more strongly associated with HF incidence rather than HF prognosis. Accordingly, continued efforts to reduce cardiotoxicity in cHL treatment are warranted and supported by the findings in this study.

### Time since cHL inversely associated with rate of lymphoma-related deaths

The mortality observed among patients with previous cHL was driven mainly by deaths related to the cHL diagnosis, especially when the index HF event within 10 years after cHL diagnosis. This may reflect a “depletion of susceptibles” effect, whereby the patients most vulnerable to their cancer die early, leaving a relatively healthier subset with more favorable long-term cHL prognosis.^22^ It is also conceivable that the occurrence of HF limited oncologic treatment opportunities, potentially contributing to early deaths, although this cannot be verified with the present data.

### Cause-specific mortality patterns

In the present study, the underlying cause of death was attributed to CV disease in 1 in 4 deaths among patients with cHL and in 42% of comparators during follow-up. In Sweden, it is the patient’s regular physician or the last physician to attend to the patient that reports the underlying and contributing causes of death to the cause of death register. The concordance between the cause of death register and patient records has been reported as 77% overall and up to 88% for deaths due to CV disease.^19^^,^^23^^,^^24^ The distribution of causes of death in this study is comparable to a study by Mubarik et al^25^, who reported that 28% of all deaths in Western Europe and 32% of deaths in Sweden between 1990 and 2021 could be attributed to CV disease. Taking into consideration the relatively high median age (73 years) in the present study, the higher occurrence of death from CV causes among comparators is to be expected as CV disease incidence increases with age.^26^

### Cardiovascular-specific prognosis following HF

Although the pathophysiology of HF in patients with cHL may differ from that of the general population—possibly reflecting myocardial injury from anthracyclines, radiation-induced fibrosis, or microvascular dysfunction rather than ischemic remodeling or hypertensive cardiomyopathy—our findings suggest that these differences do not translate into an overall markedly poorer CV prognosis once HF has developed.^27^^,^^28^ In the present study, there was no difference in CV death between patients with cHL and comparators, but comparators were more likely to have chronic or acute coronary syndrome at baseline. It is possible that certain clinical features of cancer treatment-related HF, such as degree of reduced ejection fraction or nonischemic etiology, confer a comparable—or in some cases slightly more favorable—prognosis once patients enter specialized HF care.^29^ For example, in a Milan-based prospective study investigating incidence of cardiotoxicity and effectiveness of traditional HF therapy among 2,625 cancer patients treated with anthracyclines, prompt initiation of HF therapy induced full or partial remission in 81% of cases.^30^

The pronounced increase in CV mortality beyond 10 years after cHL is likely a reflection of change in the competing risk landscape, rather than a true surge in CV hazard, although these findings should be considered exploratory considering the small number of events in each subgroup. In line with previous evidence, the present study shows that individuals who survived a decade or more after cHL before being diagnosed with HF had a substantially lower risk of lymphoma-related death, and CV causes constituted a greater proportion of total mortality.^31^ On the other hand, the pattern of increasing all-cause and CV-specific mortality in patients with ≥10 years between cHL and HF may also indicate delayed emergence of treatment-related disease from mediastinal radiotherapy, which often manifests clinically years to decades after exposure.^32^, ^33^, ^34^, ^35^ Such late effects may give rise to a distinct phenotype of HF which is potentially more ischemic, valvular, or restrictive in nature compared with the earlier-onset, predominantly anthracycline-associated HF observed in patients closer to their cHL treatment.^36^ However, this could not be confirmed in the present study, as most HF diagnoses were unspecified. Related to this, a Swedish study of trends in CV mortality among patients with cHL treated before 2006 found that the highest excess risk occurred in those diagnosed before the mid-1980s, with a subsequent decline predicted for later treatment eras.^37^ This downward trend has since been confirmed in a large U.S. population-based study including patients diagnosed between 1983 and 2015.^11^ However, this decline in CV mortality cannot only be explained by reduced incidence; in another Swedish study, the excess incidence of diseases of the circulatory system among patients with cHL decreased in the period of 1985 to 1994 but then plateaued and remained steady until 2013 (end of study).^38^ Taken together, these observations suggest that the excess CV mortality historically observed in patients with cHL may have diminished over time—possibly reflecting both improved recognition and management of CV risk factors, advances in HF and ischemic heart disease care, and ongoing refinements in lymphoma treatment to reduce cardiotoxicity. The apparent no-difference in CV-specific mortality between groups in the overall analysis should be interpreted with caution, as the strong competing risk from lymphoma-related death in the early period may mask an underlying excess CV mortality. To the extent that this finding reflects true comparability, it may also be a demonstration of general improvements in CV risk factor management and outcomes among contemporary cHL populations. Thus, while overall CV mortality may have improved in modern cHL cohorts, very long-term survivors may still carry a sustained or re-emerging burden of late cardiotoxic sequelae.

### Study Limitations

The key strength of this study is its novelty; to our knowledge, it is the first to assess outcomes of HF among patients with a previous cHL compared with lymphoma-free comparators, providing complementary evidence to prior work on CV late effects in this population. Another strength is the population-based setting, offering generalizability to patients in the real-world. Several limitations should also be acknowledged. First, HF diagnoses were identified through hospital admissions or specialty care outpatient clinics, so cases managed solely in primary care settings may have been missed, potentially biasing toward more severe disease. Second, all patients with cHL were included, irrespective of whether they had a record of any treatment. This meant that for a part of the population, detailed information on lymphoma treatment, including cumulative anthracycline dose, radiation field, and treatment intent, was unavailable. However, virtually all patients with cHL are expected to have received some type of oncological treatment, almost always including anthracyclines. Related to this, reliable information on relapse was also not available, which may especially have impacted the cause-specific <2 and ≥ 2 to 10 year analyses. Third, data on HF phenotype, left ventricular ejection fraction, and HF-directed pharmacotherapy were lacking, precluding mechanistic or therapeutic interpretation. Additionally, clinical information such as smoking status and body mass index was not available. Although these factors affect HF risk and/or prognosis, they are not necessarily considered strong confounders (ie, they do not affect both the exposure and the outcome) in this study.

## Conclusions

HF developing after cHL diagnosis was associated with a lower overall survival compared to HF in lymphoma-free patients and was mainly driven by lymphoma-specific mortality. CV-specific mortality was similar between groups, except in patients with HF diagnosis occurring a decade or more after cHL who had a higher CV-specific mortality than comparators. These exploratory findings should be interpreted cautiously due to the strong competing risk of lymphoma-related death that may obscure the true CV hazard, but could raise the hypothesis that the higher CV-specific mortality in patients with cHL, that has previously been reported, may primarily reflect increased incidence of HF rather than a more aggressive or treatment-resistant HF phenotype.

Perspectives **COMPETENCY IN MEDICAL KNOWLEDGE:** Among patients with HF, a history of cHL was associated with higher all-cause mortality than matched lymphoma-free comparators. The increased mortality rate for the cHL group was driven mainly by lymphoma-related deaths and in an explorative analysis of CV-specific death there was no difference between the groups overall. However, when more than 10 years elapsed between cHL diagnosis and HF, patients in the cHL group had a higher CV-specific mortality than comparators possibly highlighting the impact of late treatment effects. These findings underscore the importance of long-term CV surveillance in patients with a history of cHL.

**TRANSLATIONAL OUTLOOK:** Future studies in larger and more deeply phenotyped populations are needed to characterize HF subtypes after lymphoma treatment, including the contribution of prior anthracycline exposure and mediastinal radiotherapy, and to determine whether very long-term survivors represent a distinct CV risk group that may benefit from tailored surveillance or management strategies.

## Funding support and author disclosures

This study was supported by grants from the Nordic Cancer Union (project number A16008) and Åke Wiberg’s Foundation (project numbers: M23-0035 and M24-0165). The authors have reported that they have no relationships relevant to the contents of this paper to disclose.

## References

[1] Silverdal J., Sjöland H., Bollano E., Pivodic A., Dahlström U., Fu M. (2020). Prognostic impact over time of ischaemic heart disease vs. non-ischaemic heart disease in heart failure. ESC Heart Fail.

[2] van Nimwegen F.A., Schaapveld M., Janus C.P.M. (2015). Cardiovascular disease after Hodgkin lymphoma treatment: 40-year disease risk. JAMA Intern Med.

[3] Aleman B.M.P., van den Belt-Dusebout A.W., De Bruin M.L. (2007). Late cardiotoxicity after treatment for Hodgkin lymphoma. Blood.

[4] Baech J., Hansen S.M., Lund P.E. (2018). Cumulative anthracycline exposure and risk of cardiotoxicity; a Danish nationwide cohort study of 2440 lymphoma patients treated with or without anthracyclines. Br J Haematol.

[5] Lefrak E.A., Pitha J., Rosenheim S., Gottlieb J.A. (1973). A clinicopathologic analysis of adriamycin cardiotoxicity. Cancer.

[6] Baech J., El-Galaly T.C., Entrop J.P. (2024). Congestive heart failure after anthracycline-containing treatment for Hodgkin lymphoma: a Swedish matched cohort study. EJHaem.

[7] Godtfredsen S.J., Yonis H., Baech J. (2024). Risk of cardiovascular disease in patients with classical hodgkin lymphoma: a Danish nationwide register-based cohort study. Eur J Haematol.

[8] Borchmann P., Ferdinandus J., Schneider G. (2024). Assessing the efficacy and tolerability of PET-guided BrECADD versus eBEACOPP in advanced-stage, classical Hodgkin lymphoma (HD21): a randomised, multicentre, parallel, open-label, phase 3 trial. Lancet.

[9] Herrera A.F., LeBlanc M., Castellino S.M. (2024). Nivolumab+AVD in advanced-stage classic Hodgkin’s lymphoma. N Engl J Med.

[10] Boyne D.J., Mickle A.T., Brenner D.R. (2018). Long-term risk of cardiovascular mortality in lymphoma survivors: a systematic review and meta-analysis. Cancer Med.

[11] Lu Z., Teng Y., Ning X., Wang H., Feng W., Ou C. (2022). Long-term risk of cardiovascular disease mortality among classic Hodgkin lymphoma survivors. Cancer.

[12] Henson K.E., Reulen R.C., Winter D.L. (2016). Cardiac mortality among 200 000 five-year survivors of cancer diagnosed at 15 to 39 years of age. Circulation.

[13] Baech J., Husby S., Trab T. (2024). Cardiovascular diseases after high-dose chemotherapy and autologous stem cell transplant for lymphoma: a Danish population-based study. Br J Haematol.

[14] Felker G.M., Thompson R.E., Hare J.M. (2000). Underlying causes and long-term survival in patients with initially unexplained cardiomyopathy. N Engl J Med.

[15] Mariotto A.B., Noone A.M., Howlader N. (2014). Cancer survival: an overview of measures, uses, and interpretation. JNCI Monographs.

[16] Ekström Smedby K., Eloranta S., Wästerlid T. (2024). The National Swedish Lymphoma register - a systematic validation of data quality. Acta Oncol.

[17] Ludvigsson J.F., Almqvist C., Bonamy A.K.E. (2016). Registers of the Swedish total population and their use in medical research. Eur J Epidemiol.

[18] Heide-Jørgensen U., Adelborg K., Kahlert J., Sørensen H.T., Pedersen L. (2018). Sampling strategies for selecting general population comparison cohorts. Clin Epidemiol.

[19] Brooke H.L., Talbäck M., Hörnblad J. (2017). The Swedish cause of death register. Eur J Epidemiol.

[20] Wallerstedt S.M., Wettermark B., Hoffmann M. (2016). The first decade with the Swedish prescribed drug register - a systematic review of the output in the scientific literature. Basic Clin Pharmacol Toxicol.

[21] Ludvigsson J.F., Svedberg P., Olén O., Bruze G., Neovius M. (2019). The longitudinal integrated database for health insurance and labour market studies (LISA) and its use in medical research. Eur J Epidemiol.

[22] Hernán M.A. (2010). The hazards of hazard ratios. Epidemiology.

[23] Johansson L.A., Björkenstam C., Westerling R. (2009). Unexplained differences between hospital and mortality data indicated mistakes in death certification: an investigation of 1,094 deaths in Sweden during 1995. J Clin Epidemiol.

[24] Eriksson A., Stenlund H., Ahlm K. (2013). Accuracy of death certificates of cardiovascular disease in a community intervention in Sweden. Scand J Public Health.

[25] Mubarik S., Naeem S., Shen H. (2024). Population-level distribution, risk factors, and burden of mortality and disability-adjusted life years attributable to major noncommunicable diseases in Western Europe (1990-2021): ecological analysis. JMIR Public Health Surveill.

[26] Tian F., Chen L., Qian Z. (2023). Ranking age-specific modifiable risk factors for cardiovascular disease and mortality: evidence from a population-based longitudinal study. EClinicalMedicine.

[27] Rasmussen M., Prado A., Hominal M.A. (2023). Global variations in heart failure etiology, management, and outcomes. JAMA.

[28] Armenian S.H., Armstrong G.T., Aune G. (2018). Cardiovascular disease in survivors of childhood cancer: insights into epidemiology, pathophysiology, and prevention. J Clin Oncol.

[29] Liang M., Bian B., Yang Q. (2022). Characteristics and long-term prognosis of patients with reduced, mid-range, and preserved ejection fraction: a systemic review and meta-analysis. Clin Cardiol.

[30] Cardinale D., Colombo A., Bacchiani G. (2015). Early detection of anthracycline cardiotoxicity and improvement with heart failure therapy. Circulation.

[31] Biccler J.L., Glimelius I., Eloranta S. (2019). Relapse risk and loss of lifetime after modern combined modality treatment of young patients with Hodgkin lymphoma: a Nordic lymphoma epidemiology group study. J Clin Oncol.

[32] Heidenreich P.A., Schnittger I., Strauss H.W. (2007). Screening for coronary artery disease after mediastinal irradiation for Hodgkin’s disease. J Clin Oncol.

[33] Reinders J.G., Heijmen B.J., Olofsen-van Acht M.J., van Putten W.L., Levendag P.C. (1999). Ischemic heart disease after mantlefield irradiation for Hodgkin’s disease in long-term follow-up. Radiother Oncol.

[34] Heidenreich P.A., Hancock S.L., Vagelos R.H., Lee B.K., Schnittger I. (2005). Diastolic dysfunction after mediastinal irradiation. Am Heart J.

[35] Heidenreich P.A., Hancock S.L., Lee B.K., Mariscal C.S., Schnittger I. (2003). Asymptomatic cardiac disease following mediastinal irradiation. J Am Coll Cardiol.

[36] Cardinale D., Colombo A., Lamantia G. (2010). Anthracycline-induced cardiomyopathy. J Am Coll Cardiol.

[37] Eloranta S., Lambert P.C., Sjöberg J., Andersson T.M.L., Björkholm M., Dickman P.W. (2013). Temporal trends in mortality from diseases of the circulatory system after treatment for Hodgkin lymphoma: a population-based cohort study in Sweden (1973 to 2006). J Clin Oncol.

[38] Weibull C.E., Björkholm M., Glimelius I. (2019). Temporal trends in treatment-related incidence of diseases of the circulatory system among Hodgkin lymphoma patients. Int J Cancer.

