# Supplementary material for: Mortality in Patients With Hodgkin Lymphoma and Heart Failure: A Swedish Population-Based Study
Source: JACC Adv. 2026 Jul 22;5(8):103058. doi: 10.1016/j.jacadv.2026.103058 (PMC13425852; doi:10.1016/j.jacadv.2026.103058)
Supplement: Supplemental Material [file mmc1.pdf]

Supplementary material

Details on statistical methods

Model selection

Flexible parametric survival models [1, 2] were used throughout, as they provide hazard ratios and 95% confidence intervals corresponding to those from a standard Cox model, while also providing seamless incorporation time-dependent effects (when needed) and ease of estimating survival probabilities and similar absolute measures. All modelling was performed in Stata using the packages merlin [3] and stpm3 [2, 4]

Model complexity (dfs for the baseline hazard and time-dependent effect) was guided by the Akaike and Bayesian information criteria (AIC and BIC). The proportional hazards assumption was evaluated using Schoenfeld residuals using the Grambsch-Therneu test.

The non-linear effects of age and year were modelled using restricted cubic splines with four degrees of freedom, resulting in three internal knots and two boundary knots. Knots were placed at equally spaced centiles of the distribution of each respective variable. For age this resulted in: 22, 63, 73, 80, 94, and for year: 2000, 2011, 2017, 2020, 2022. The spline variables were orthogonalized using Gram-Schmidt orthogonalization.

Standardization

Standardized overall survival curves were predicted from an adjusted model where the proportional hazards assumption was relaxed (two df for the time-dependent effect) and further including an interaction term between cHL status and sex and age, respectively, to allow for effect modification. Standardization was done first over all adjustment variables and secondly over calendar year and source of HF diagnosis (yielding age- and sex-specific curves).

Similarly, covariate-pattern specific predictions of cumulative risk of cardiovascular death were performed and standardized over the distribution of calendar year of index HF and contact type in the study population.

Sensitivity analysis

Within the cHL group, the effect of time to index HF (2 to <10, and ≥10 years, compared with <2 years) adjusted for lymphoma-related factors was further evaluated using flexible parametric models (three df for baseline rate). The model was adjusted for age at HL diagnosis, sex, year of HL, Ann Arbor stage (limited, IA-IIA/advanced, IIB-IVB), chemotherapy treatment group, radiotherapy (yes/no). Missing values on treatment and radiotherapy were included as categories, and an indicator for having missing information was additionally included in the models.

References

1. Royston P, Parmar MKB (2002) Flexible parametric proportional-hazards and proportional-odds models for censored survival data, with application to prognostic modelling and estimation of treatment effects. Stat Med 21(15):2175–2197. https://doi.org/10.1002/sim.1203

2. Royston P, Lambert PC (2011) Flexible Parametric Survival Analysis Using Stata: Beyond the Cox Model. StataCorp LLC

3. Crowther MJ (2020) merlin—A unified modeling framework for data analysis and methods development in Stata. The Stata Journal: Promoting communications on statistics and Stata 20(4):763–784. https://doi.org/10.1177/1536867X20976311

4. Lambert P (2023) stpm3. https://pclambert.net/software/stpm3.html. Accessed 3 Jun 2026

sFigure 1: Boxplot showing the distribution of the time interval between classical Hodgkin lymphoma (cHL) and subsequent heart failure (HF) diagnosis among the 257 patients with cHL. The box represents the interquartile range, the horizontal line within the box denotes the median, and the whiskers extend to 1.5 times the interquartile range.

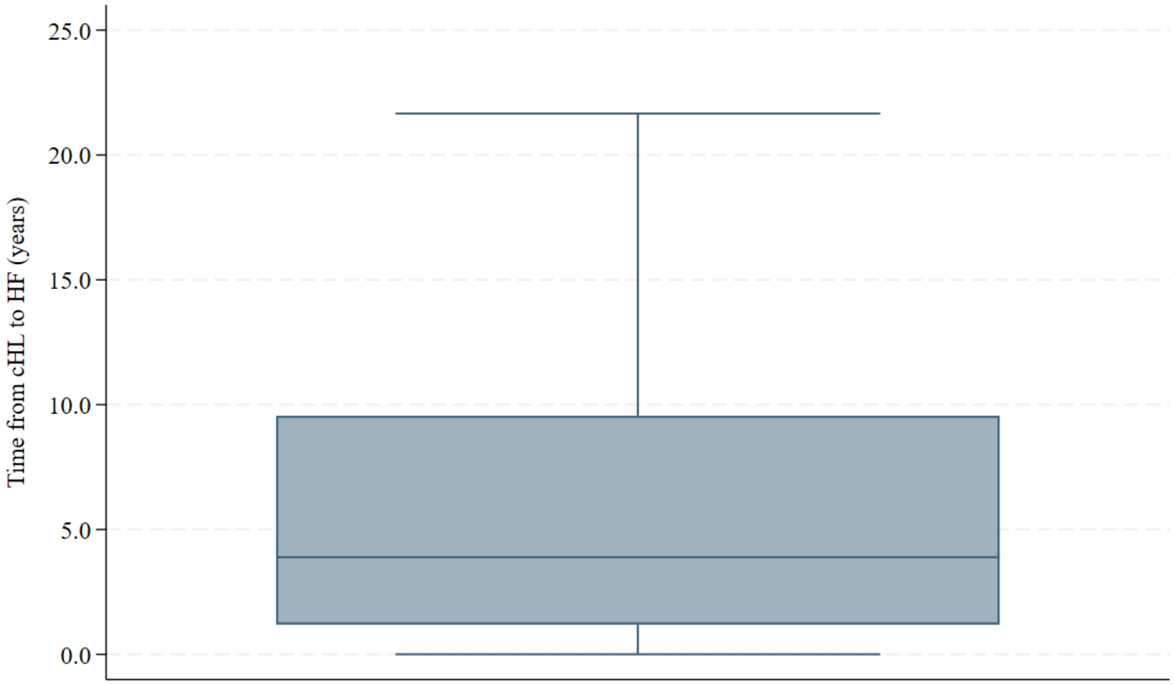

sFigure 2: Overall survival after incident heart failure (HF) among patients previously diagnosed with classical Hodgkin lymphoma (cHL) and matched lymphoma-free comparators. Estimated using the Kaplan-Meier method. P-value from log rank test of equality between survival functions.

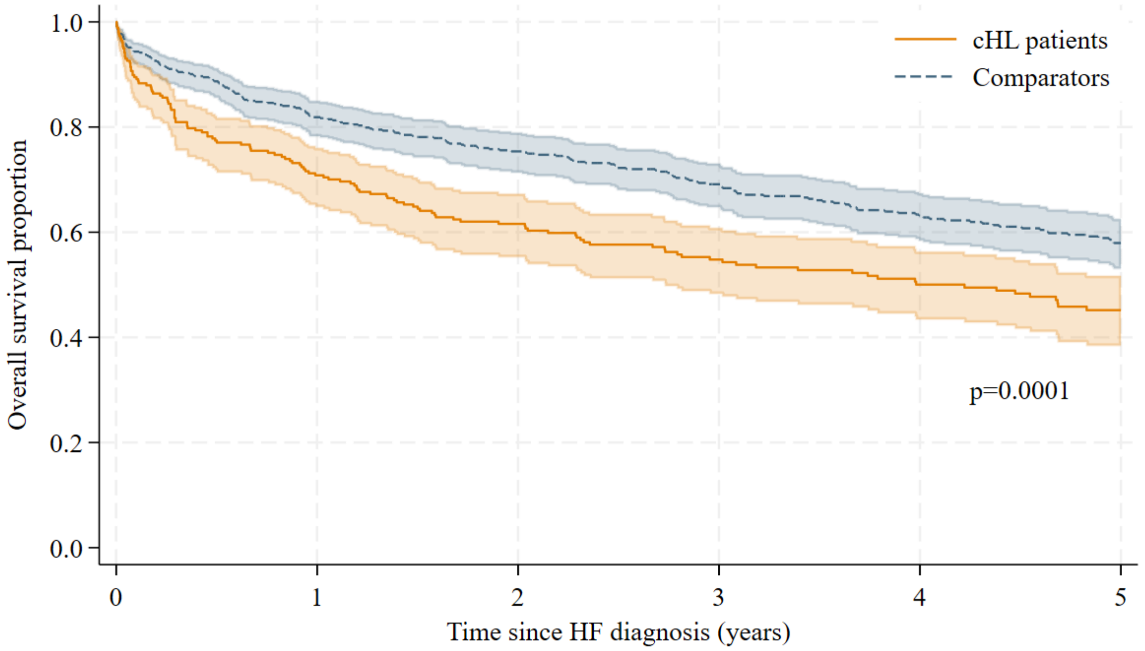

**At risk**	0	1	2	3	4	5
cHL patients	514	419	352	277	225	183
Comparators	257	182	143	111	90	65

sFigure 3: Time-dependent hazard ratios (HRs*) with 95% confidence intervals (CIs) comparing all-cause mortality rates (left panel) and cardiovascular-specific mortality rates (right panel) among patients with heart failure (HF), comparing those previously diagnosed with classical Hodgkin lymphoma (cHL) to matched lymphoma-free comparators.

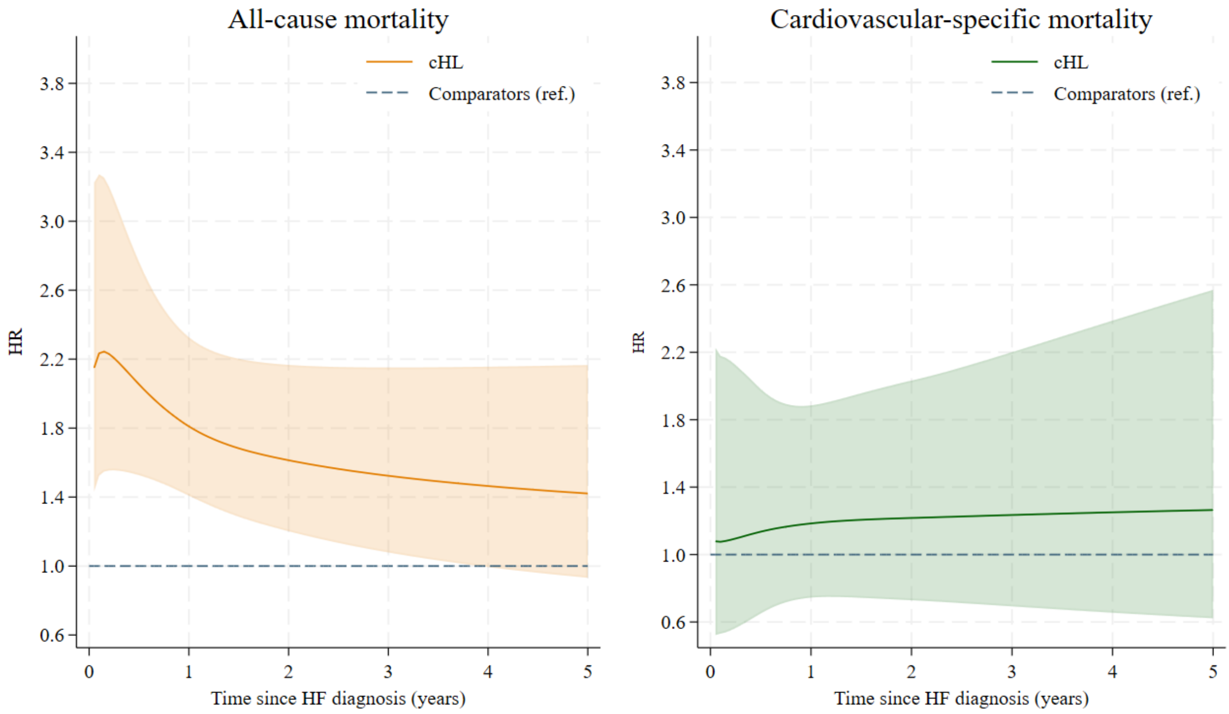

** Estimated from flexible parametric non-proportional hazards models (with three degrees of freedom (df) for the baseline rate and two for the time-dependent effect), adjusted for all matching variables: Age and year of HF diagnosis, sex, and source of HF (in-/outpatient register). Age and year were modelled assuming non-linear effects, using restricted cubic splines with four df.*

sFigure 4: Cumulative risk and estimates (table) of cardiovascular death, accounting for the competing risk of death from other causes, after incident heart failure (HF) diagnosis among patients previously diagnosed with classical Hodgkin lymphoma (cHL) and matched lymphoma-free comparators. Estimated using the Aalen-Johansen method.

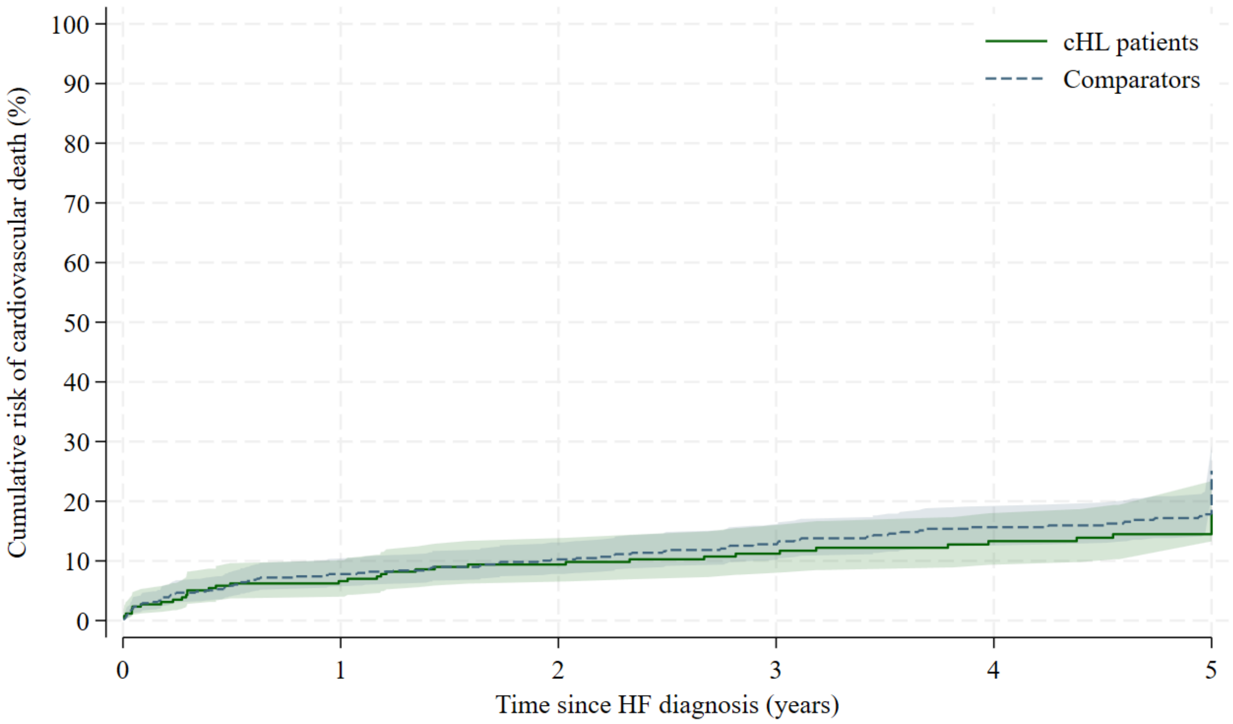

Point-wise estimates of cardiovascular-specific death (in %) with 95% confidence intervals (CIs)			
	**1 year**<br>Prob (95% CI)	**2 years**<br>Prob (95% CI)	**5 years**<br>Prob (95% CI)
**cHL patients**	6.61 (4.01-10.1)	9.83 (6.56-13.9)	14.5 (10.3-19.4)
**Comparators**	7.80 (5.69-10.3)	10.3 (7.80-13.1)	17.8 (14.4-21.5)

sFigure 5: Standardized cumulative risk of cardiovascular death among males (top panel) and females (bottom panel) aged 60 (left) and 80 (right) at heart failure (HF) diagnosis, in patients with a previous classical Hodgkin lymphoma (cHL) and matched comparators. Point-wise estimates with uncertainty are given in the accompanying table below.

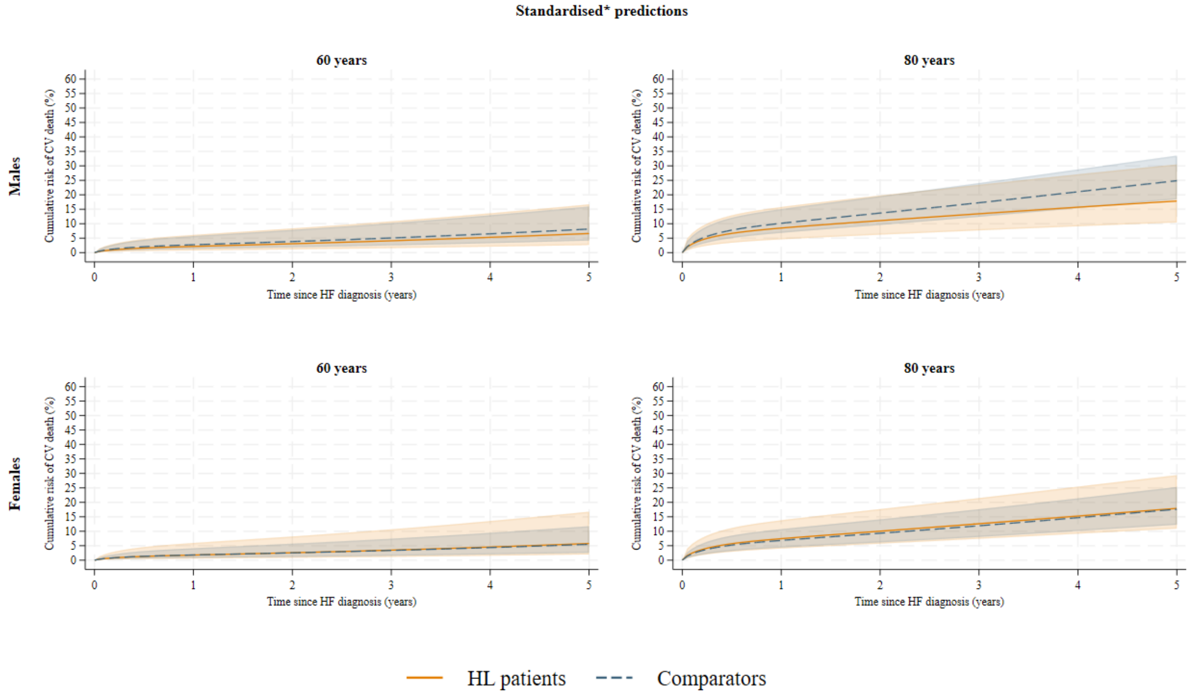

Point-wise cumulative risk estimates (in %) with 95% confidence intervals (CIs).

	Sex	Age at diagnosis	**1 year** Prob (95% CI)	**2 years** Prob (95% CI)	**5 years** Prob (95% CI)
**cHL patients**	Males	60 years	2.2 (0.8-6.2)	3.1 (1.1-8.4)	6.7 (2.6-16.7)
		80 years	8.5 (4.6-15.8)	11.1 (6.2-19.8)	17.8 (10.4-30.5)
	Females	60 years	1.8 (0.5-6.0)	2.6 (0.8-8.2)	5.76 (2.0-16.8)
		80 years	7.4 (4.0-13.8)	10.0 (5.7-17.7)	17.9 (10.9-29.4)
**Comparators**	Males	60 years	2.7 (1.3-5.6)	3.8 (1.9-7.9)	8.2 (4.2-16.0)
		80 years	10.1 (6.8-15.1)	13.7 (9.6-19.5)	24.9 (18.4-33.5)
	Females	60 years	1.8 (0.8-4.14)	2.5 (1.1-5.7)	5.5 (2.6-11.8)
		80 years	6.8 (4.4-10.7)	9.32 (6.2-14.1)	17.6 (12.3-25.3)

** Predicted from a flexible parametric non-proportional hazards model (with three degrees of freedom (df) for the baseline rate and two for the time-dependent effect), adjusted for all matching variables: Age and year of index HF, sex, and source of HF (in-/outpatient register). Age and year were modelled assuming non-linear effects, using restricted cubic splines with four df. The effect of HL was allowed to vary between sexes and ages by including an interaction term. Standardized over calendar year and source of HF diagnosis.*

sFigure 6: Hazard ratio (HR) with 95% confidence interval (CI) for the effect of time interval between classical Hodgkin lymphoma (cHL) and heart failure (HF) on all-cause (left panel) and cardiovascular-specific (right panel) mortality in patients with cHL relative to matched comparators. Time interval modelled using a restricted cubic spline with four degrees of freedom.

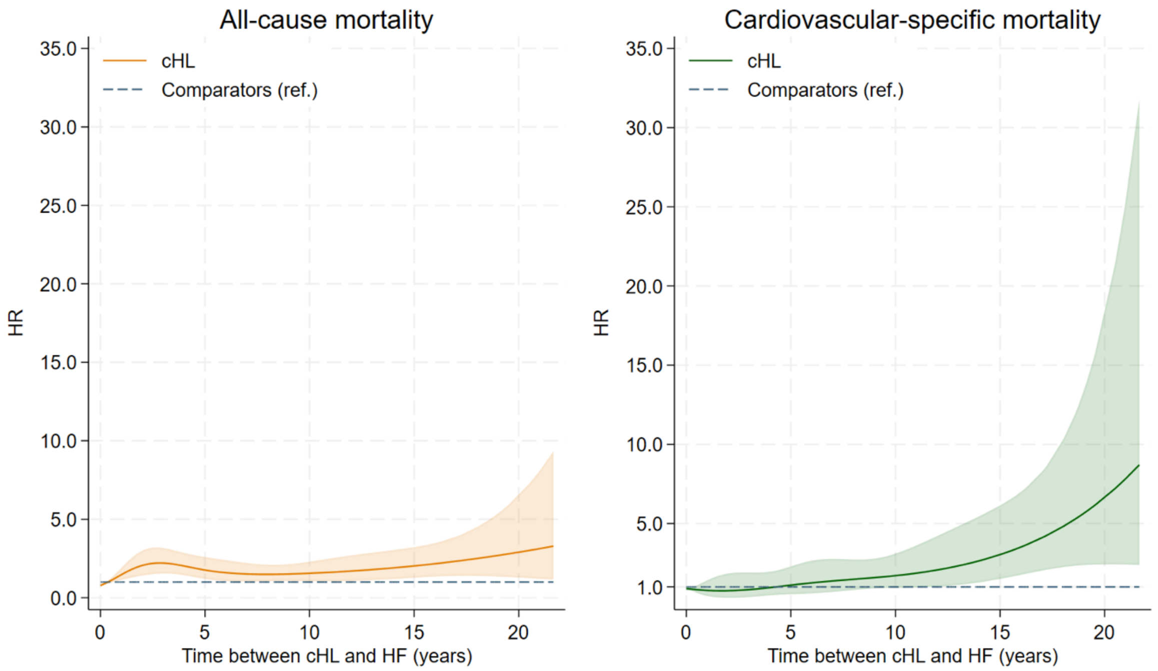

sTable 1: Anatomical Therapeutic Chemical (ATC), World Health Organization's International Classification of Diseases (ICD) versions 9 and 10, and ICD for Oncology version 3 codes used to define classical Hodgkin lymphoma, heart failure, pre-existing conditions and cardiovascular death in the population**.**

Condition/characteristic	Definition	Register source	Available for
**Baseline characteristics**			
**Classical Hodgkin lymphoma**	**ICD-O3:** 510, 520, 530, 540, 550	SLR	Only cHL
**Heart failure**	**ICD-9**: 428, 429<br>**ICD-10**: DI110, DI130, DI132, DI255, DI420, DI428, DI429, DI50.x	NPR	Both groups
**Hypertension**	**ICD-9**: 401B, 401X, 402, 403, 404<br>**ICD-10**: DI10, DI11, DI12, DI13, DI15	NPR	Both groups
**Chronic kidney disease**	**ICD-9**: 403, 404, 580, 581, 582, 583, 584, 250D, 719A, 587<br>**ICD-10**: DE102, DE112, DE132, DE142, DI120, DN18, DN19, DI120, DQ612, DQ613, DQ615, DQ619	NPR	Both groups
**Peripheral artery disease**	**ICD-9**: 440<br>**ICD-10**: DI70.x, DI71.x, DI73.x	NPR	Both groups
**Ischemic stroke**	**ICD-9**: 433, 434, 435, 436, 437<br>**ICD-10**: DI63.x, DI64.x, DG458, DG459	NPR	Both groups
**Diabetes mellitus**	**ICD-9**: 250, 257C<br>**ICD-10**: DE10.x, DE11.x, DE13.x, DE14.x<br>**ATC**: A10AB01, A10AB04, A10AB05, A10AB06, A10AC01, A10AD01, A10AD04, A10AD05, A10AD06, A10AE04, A10AE05, A10AE06, A10AE56, A10BA02, A10BB07, A10BB09, A10BB12, A10BD07, A10BD13, A10BD15, A10BD16, A10BD19, A10BD20, A10BD21, A10BG, A10BH01, A10BH02, A10BH03, A10BH04, A10BH05, A10BJ02, A10BJ05, A10BJ06, A10BK01, A10BK02, A10BK03, A10BX02	NPR and PDR	Both groups
**Atrial fibrillation**	**ICD-9**: 427<br>**ICD-10**: DI48.x	NPR	Both groups
**Acute coronary syndrome**	**ICD-9**: 410, 411<br>**ICD-10**: DI21.x, DI200	NPR	Both groups

**Chronic coronary syndrome**	**ICD-9**: 412, 413, 414<br>**ICD-10**: DI208, DI209, DI249, DI250, DI258	NPR	Both groups
**Education level**	Education level	LISA	Both groups
**Causes of death**			
**Cardiovascular**	**ICD-10**: DI.x	CODR	Both groups
**Lymphoma**	**ICD-10:** DC81.x–DC86.x, DC93.x	CODR	Both groups
**Non-lymphoma**	**ICD-10**: All other malignant neoplasms (DC00–DC80, DC88–DC97) including leukemias (DC90*, DC91.x, DC92.x, DC93.x), and selected neoplasms of uncertain or unknown behavior (DD13.x, DD37.x, DD41.x, DD46.x, DD47.x)	CODR	Both groups
**Chronic obstructive pulmonary disease**	**ICD-10:** DJ43.x, DJ44.x	CODR	Both groups
**Other lung disease**	**ICD-10:** J43.x, J44.x	CODR	Both groups
**Infection**	**ICD-10**: DA00-DA99, DB00-DB99, DJ09-DJ18, DK65	CODR	Both groups
**Diabetes mellitus**	**ICD-10**: DE10-DE14	CODR	Both groups
**Alzheimer's disease and other dementias**	**ICD-10:** DF0.x, DG3.x	CODR	Both groups
**Lymphoma specific**			
**ECOG performance status**	Performance status according to WHO/ECOG: 0 = Asymptomatic; 1 = Symptomatic but completely ambulatory; 2 = Symptomatic, <50% in bed during the day; 3 = Symptomatic, >50% in bed, but not bedbound; 4 = Bedbound; 9 = Missing	SLR	Only cHL
**Ann-Arbor stage**	Combined from two variables: a staging variable indicating lymph node involvement (stage I-IV) and a B-symptom variable indicating presence/absence.	SLR	Only cHL
**Serum lactate dehydrogenase**	Indicates LDH level at diagnosis, dichotomized as normal/abnormal based on reference values of sld > 3.5 (age 18-70), sld > 4.3 (age > 70)	SLR	Only cHL
**Bulky disease**	Presence of bulky disease at diagnosis	SLR	Only cHL
**Chemotherapy**	Contains all possible chemotherapy regimens. Collapsed into: BEACOPP including both escalated BEACOPP, BEACOPPDac,	SLR	Only cHL

	regular BEACOPP, and BrECADD; A(B)VD; CHOP-like including both (R)-CHOP and (R)-CHOEP; Other including bendamustin, CEOP, chlorambucil, cyclophosphamide, DHAP, vadriac, and MOPP regimens		
**Cumulative anthracycline dose**	Calculated from assumed normal doses of chemotherapy regimens and number of cycles recorded. Assumed normal doses: A(B)VD: 50 mg/m^2 per cycle, BEACOPP: mg/m^2 per cycle, BEACOPPesc mg/m^2 per cycle, BrECADD 160 mg/m^2 total, (R)-CHO(E)P: mg/m^2 per cycle, MAXI-CHOP: mg/m^2 per cycle, MINI-CHOP 25 mg/m^2 per cycle,	SLR	Only cHL
**Radiotherapy**	Recorded as yes/no by treating physician	SLR	Only cHL

.x: Includes all diagnoses in the subcategory. Abbreviations: SLR: Swedish Lymphoma Register NPR: National Patient Register, PDR: Prescribed drug register, CODR: Cause of death Register, LISA: Longitudinal Integrated Database for Health Insurance and Labor Market Studies, ABVD: doxorubicin-bleomycin-vinblastine-dacarbacine; BEACOPP: bleomycine-etoposide-doxorubicin-cyclophosphamide-vincristine-procarbazine-prednisolone; CHOP: doxorubicin-cyclophosphamide-vincristine-prednisone. CEOP: cyclophosphamide-etoposide-vincristine, LVP: L-asparginase-vincristine-prednisolone, DHAP: dexamethasone-cytarabine-cisplatin, MOPP: mechlorethamine-vincristine-procarbazine-prednisone.

sTable 2: Hazard ratios (HRs) with 95% confidence intervals (CIs) as measures of relative all-cause (top) and cardiovascular-specific (bottom) mortality rates among patients with heart failure (HF) and a previous classical Hodgkin lymphoma (cHL) diagnosis, by time from cHL to HF diagnosis.

**All-cause mortality**	**HR* (95% CI)**
<2 years since cHL	1.00 (ref.)
≥2 to <10 years since cHL	1.79 (1.08-2.97)
≥10 years since cHL	3.04 (1.53-6.08)
**Cardiovascular-specific mortality**	
<2 years since cHL	1.00 (ref.)
≥2 to <10 years since cHL	4.50 (1.24-16.3)
≥10 years since cHL	34.6 (7.50-160)

** Estimated from flexible parametric proportional hazards models with three degrees of freedom (df) for the baseline hazard rate adjusted for age and year of cHL diagnosis, sex, stage (limited/advanced), and treatment with chemo- and radiotherapy for cHL. Age and year were modelled assuming non-linear effects, using restricted cubic splines with four df. Missing values on stage, chemo- and radiotherapy were included in the model with an indicator for missing information additionally added.*

STROBE Statement—checklist of items that should be included in reports of observational studies

	Item No.	Recommendation	Page No.	Relevant text from manuscript
**Title and abstract**	1	(*a*) Indicate the study's design with a commonly used term in the title or the abstract	1	Title: "(…) A Swedish population-based study" indicates cohort study design. Abstract states: "register-based matched cohort study"
		(*b*) Provide in the abstract an informative and balanced summary of what was done and what was found	2 (*Abstract*)	Abstract provides background, objectives, methods (matched cohort, 257 cHL vs. 514 comparators, flexible parametric models), results (HR=1.81, CV-specific HR=1.17; HR=3.13 for ≥10 years), and conclusions on all-cause and CV-specific mortality.
**Introduction**				
Background/rationale	2	Explain the scientific background and rationale for the investigation being reported	4 (*Introduction*)	Introduction describes HF risk after cHL, CV toxicity as leading cause

				of morbidity/mortality, and the knowledge gap about survival conditional on HF development.
Objectives	3	State specific objectives, including any prespecified hypotheses	4 (*Introduction*)	"The aim of this study was to assess all-cause mortality following HF in patients with a prior cHL diagnosis compared to matched lymphoma-free comparators."
**Methods**				
Study design	4	Present key elements of study design early in the paper	5 (*Material and methods*)	Stated explicitly in the first sentence of the Methods section.
Setting	5	Describe the setting, locations, and relevant dates, including periods of recruitment, exposure, follow-up, and data collection	5 (*Material and methods*)	Sweden; nationwide register-based study using LymphomaBase (Swedish Lymphoma Register 2000–2023), National Patient Register, National Cause of Death Register, and other national health registers. Recruitment 2000-2022; follow-up

				ended December 31, 2023 or maximum 5 years.
Participants	6	(*a*) *Cohort study*—Give the eligibility criteria, and the sources and methods of selection of participants. Describe methods of follow-up	5 (*Material and methods*)	Eligibility: patients aged ≥18 years at cHL diagnosis (n=4,049) from the Swedish Lymphoma Register with incident HF after cHL diagnosis; matched 1:2 to lymphoma-free comparators on sex, birth year, year of HF, and contact type (in-/outpatient). Main exclusion criteria were HF prior to cHL diagnosis.
		(*b*) *Cohort study*—For matched studies, give matching criteria and number of exposed and unexposed	5-6 (*Material and methods*)	Matched 1:2 on sex, birth year, year of HF, and contact type. If matching unsuccessful, birth year and HF year tolerance relaxed stepwise in 2-year increments up to ±8 years. Final cohort: 257 cHL patients, 514 comparators.

Variables	7	Clearly define all outcomes, exposures, predictors, potential confounders, and effect modifiers. Give diagnostic criteria, if applicable	6 (*Material and methods*) and *sTable 1*	Outcome: all-cause death (primary); CV-specific death (exploratory). Exposure: prior cHL diagnosis. Confounders/matching variables: sex, age at HF, calendar year of HF, contact type. Comorbidities (hypertension, CKD, AF, ACS, etc.) retrieved from National Patient Register and Drug Register. HF defined by ICD-9/10 codes (sTable 1).
Data sources/ measurement	8*	For each variable of interest, give sources of data and details of methods of assessment (measurement). Describe comparability of assessment methods if there is more than one group	*sTable 1*	
Bias	9	Describe any efforts to address potential sources of bias	7 (*Material and methods*) and Supplementary Material *Details on*	Advances survival analyis using flexible parametric models adjusted for main confounders.

			statistical methods	
Study size	10	Explain how the study size was arrived at	5 (*Material and methods*)	LymphomaBase (Swedish Lymphoma Register): cHL diagnosis, disease characteristics, treatment. National Patient Register (ICD-9/10): HF identification and comorbidities.

Continued on next page

Quantitative variables	11	Explain how quantitative variables were handled in the analyses. If applicable, describe which groupings were chosen and why	7 (*Material and methods*) and Supplementary Material *Details on statistical methods*	The main analyses were performed on the entire population (257 patients with cHL and HF vs. 514 comparators with HF). Exploratory analyses were performed based on time since cHL dividing the population in increments from <2 years, 2-<10 years, or ≥10 years between cHL and HF.
Statistical methods	12	(*a*) Describe all statistical methods, including those used to control for confounding	7 (*Material and methods*) and Supplementary Material *Details on statistical methods*	The study employed flexible parametric survival modeling and have described the method and approach to confounder adjustment and standardiztion in details.
		(*b*) Describe any methods used to examine subgroups and interactions	7 (*Material and methods*)	
		(*c*) Explain how missing data were addressed	NA	No variables with potential missing information was used in the analyses. Missing information for the

				descriptive statistics are indicated in *Table 1* and *Table 2*.
		(*d*) *Cohort study*—If applicable, explain how loss to follow-up was addressed	NA	
		(*e*) Describe any sensitivity analyses	7 (*Material and methods*), Supplementary Material *Details on statistical methods,* page 9 (*Results: All-cause death*)	Displaying time-varying HR and performing analyses of the primary outcome with further covariate adjustment.
**Results**				
Participants	13*	(a) Report numbers of individuals at each stage of study—eg numbers potentially eligible, examined for eligibility, confirmed eligible, included in the study, completing follow-up, and analysed	5 (*Material and methods*) and *Table 2*	Descirbes the study population in detail and in Table 2 the reader can compare the study population to the overall cHL source population.
		(b) Give reasons for non-participation at each stage	NA	
		(c) Consider use of a flow diagram	NA	
Descriptive data	14*	(a) Give characteristics of study participants (eg demographic, clinical, social) and information on exposures and potential confounders	*Table 1* and *Table 2*	Information on confounders applicable to both cases and

				comparators (*Table 1*) and lymphoma-specific information (*Table 2*).
		(b) Indicate number of participants with missing data for each variable of interest	*Table 1* and *Table 2*	
		(c) *Cohort study*—Summarise follow-up time (eg, average and total amount)	8 (*Results: Baseline characteristics*) and results tables related to each outcome	
Outcome data	15*	*Cohort study*—Report numbers of outcome events or summary measures over time	9-10 (*Results: All-cause death* and *Cause-specific death*) and results tables related to each outcome	
Main results	16	(*a*) Give unadjusted estimates and, if applicable, confounder-adjusted estimates and their precision (eg, 95% confidence interval). Make clear which confounders were adjusted for and why they were included	9 (*Results: All-cause death*), results tables related to each	

		outcome and *sFigure 2*
	(*b*) Report category boundaries when continuous variables were categorized	7 (*Material and methods: Statistical analysis*)
	(*c*) If relevant, consider translating estimates of relative risk into absolute risk for a meaningful time period	9-11 (*Results*)

Continued on next page

Other analyses	17	Report other analyses done—eg analyses of subgroups and interactions, and sensitivity analyses	9-11 (*Results*)	
**Discussion**				
Key results	18	Summarise key results with reference to study objectives	12 (*Discussion*)	
Limitations	19	Discuss limitations of the study, taking into account sources of potential bias or imprecision. Discuss both direction and magnitude of any potential bias	15 (*Discussion: Limitations*)	
Interpretation	20	Give a cautious overall interpretation of results considering objectives, limitations, multiplicity of analyses, results from similar studies, and other relevant evidence	16 (*Conclusions*)	
Generalisability	21	Discuss the generalisability (external validity) of the study results	15 (*Discussion: Limitations*)	
**Other information**				
Funding	22	Give the source of funding and the role of the funders for the present study and, if applicable, for the original study on which the present article is based		Funding sources are clearly stated in the manuscript.

*Give information separately for cases and controls in case-control studies and, if applicable, for exposed and unexposed groups in cohort and cross-sectional studies.

Note: An Explanation and Elaboration article discusses each checklist item and gives methodological background and published examples of transparent reporting. The STROBE checklist is best used in conjunction with this article (freely available on the Web sites of PLoS Medicine at http://www.plosmedicine.org/, Annals of Internal Medicine at http://www.annals.org/, and Epidemiology at http://www.epidem.com/). Information on the STROBE Initiative is available at www.strobe-statement.org.
